# Multi-omic profiling reveals associations between the gut mucosal microbiome, the metabolome, and host DNA methylation associated gene expression in patients with colorectal cancer

**DOI:** 10.1186/s12866-020-01762-2

**Published:** 2020-04-23

**Authors:** Qing Wang, Jianzhong Ye, Daiqiong Fang, Longxian Lv, Wenrui Wu, Ding Shi, Yating Li, Liya Yang, Xiaoyuan Bian, Jingjing Wu, Xianwan Jiang, Kaicen Wang, Qiangqiang Wang, Mark P. Hodson, Loïc M. Thibaut, Joshua W. K. Ho, Eleni Giannoulatou, Lanjuan Li

**Affiliations:** 1 State Key Laboratory for Diagnosis and Treatment of Infectious Diseases, The First Affiliated Hospital, School of Medicine, Zhejiang University, Hangzhou, China; 2 Collaborative Innovation Centre for Diagnosis and Treatment of Infectious Diseases, Hangzhou, China; 3 Computational Genomics Laboratory, Victor Chang Cardiac Research Institute, Sydney, Australia; 4 Freedman Foundation Metabolomics Facility, Victor Chang Innovation Centre, Victor Chang Cardiac Research Institute, Sydney, Australia; 5 School of Pharmacy, University of Queensland, Woolloongabba, QLD 4102 Australia; 6 School of Mathematics and Statistics, UNSW Sydney, Sydney, Australia; 7 Bioinformatics and Systems Medicine Laboratory, Victor Chang Cardiac Research Institute, Sydney, Australia; 8 School of Biomedical Sciences, Li Ka Shing Faculty of Medicine, The University of Hong Kong, Hong Kong, China; 9 St Vincent’s Clinical School, UNSW Sydney, Sydney, Australia

**Keywords:** Colorectal cancer, Mucosal microbiome, Metabolome, Transcriptome, DNA methylation, Butyrate

## Abstract

**Background:**

The human gut microbiome plays a critical role in the carcinogenesis of colorectal cancer (CRC). However, a comprehensive analysis of the interaction between the host and microbiome is still lacking.

**Results:**

We found correlations between the change in abundance of microbial taxa, butyrate-related colonic metabolites, and methylation-associated host gene expression in colonic tumour mucosa tissues compared with the adjacent normal mucosa tissues. The increase of genus *Fusobacterium* abundance was correlated with a decrease in the level of 4-hydroxybutyric acid (4-HB) and expression of immune-related peptidase inhibitor 16 (*PI16*), Fc Receptor Like A (*FCRLA*) and Lymphocyte Specific Protein 1 (*LSP1*). The decrease in the abundance of another potentially 4-HB-associated genus, *Prevotella 2,* was also found to be correlated with the down-regulated expression of metallothionein 1 M (*MT1M*). Additionally, the increase of glutamic acid-related family *Halomonadaceae* was correlated with the decreased expression of reelin (*RELN*). The decreased abundance of genus *Paeniclostridium* and genus *Enterococcus* were correlated with increased lactic acid level, and were also linked to the expression change of Phospholipase C Beta 1 (*PLCB1*) and Immunoglobulin Superfamily Member 9 (*IGSF9*) respectively. Interestingly, 4-HB, glutamic acid and lactic acid are all butyrate precursors, which may modify gene expression by epigenetic regulation such as DNA methylation.

**Conclusions:**

Our study identified associations between previously reported CRC-related microbial taxa, butyrate-related metabolites and DNA methylation-associated gene expression in tumour and normal colonic mucosa tissues from CRC patients, which uncovered a possible mechanism of the role of microbiome in the carcinogenesis of CRC. In addition, these findings offer insight into potential new biomarkers, therapeutic and/or prevention strategies for CRC.

## Background

Colorectal cancer (CRC) is the third most commonly diagnosed cancer and second leading cause of cancer death in both men and women globally, leading to an estimated 551,269 annual deaths worldwide [1]. Diet plays an important role in the initiation, promotion and progression of colon carcinogenesis [2]. Epidemiological surveys indicate that approximately 80% of CRC cases in Western countries are due to dietary factors [3].

The human intestine harbours a complex microbial community comprising more than 10^14^ microorganisms; it carries greater than 150 times the number of genes in the human genome [4]. Diet has been shown to have a dominant impact on the structure and composition of the gut microbiome, microbial generated metabolites and host metabolism [5, 6]. Many studies have demonstrated the effect of the gut microbiome on the pathogenesis of CRC, revealing potential pathogenic bacteria such as *Fusobacterium* as well as beneficial bacteria such as *Lactobacillales* [6–8].

Nevertheless, dietary factors have also been shown to induce epigenetic changes. The most extensively studied epigenetic mechanism is DNA methylation, which involves the transfer of methyl group to the cytosine of a CpG dinucleotide and is responsible for regulating the expression of genes related with critical pathological processes [9]. Carcinogenic gene silencing may occur due to aberrant gene-specific hypermethylation at the CpG rich region (CpG island) which is located in the promotors of the genes and interfere with transcription factor binding [10, 11].

One possible mechanism underlying the effects of diet and microbiome on CRC development is a potential interaction between the microbiome and the host, whereby the colonic metabolome is impacted, leading to a subsequent alteration in host epigenetic activity and host gene expression [12, 13]. Research on selected aspects of the microbiome, metabolome, host epigenome and host transcriptome have been carried out in human, animal and cell models [14]. For instance, the interaction between the microbiome and metabolome has been extensively studied, revealing various epigenome-modulation-related metabolites such as butyrate and folate [15, 16]. In addition, the contribution of commensal bacteria to epigenetic control in the host large intestine has been demonstrated by comparing conventional and germ-free mice [17]. Associations between the microbiome and differentially methylated genes have also been investigated in patients with ulcerative colitis [18]. The interplay between the microbiome, host transcripts related to adhesion molecules and fatty acid biosynthesis was strongly supported in one study of inflammatory bowel disease [19].

Despite the mounting evidence of a potential host-microbiome interaction, a comprehensive human study integrating all the aforementioned omics is still lacking. Thus, in this pilot study, we generated and analysed four types of omic data: the microbiome (16S rRNA sequencing; 36 pairs), the metabolome (untargeted GC/MS; 17 pairs), the host transcriptome (RNA-seq; 4 pairs) and the host epigenome (Infinium HumanMethylation850 BeadChip array; 4 pairs), as measured from paired tumour and adjacent normal colonic mucosa tissues samples obtained from CRC patients (details of the study design in Additional file 1: Figure S1).

## Results

### Comparison of microbial composition between tumour and adjacent normal tissues

**Fig. 1:**
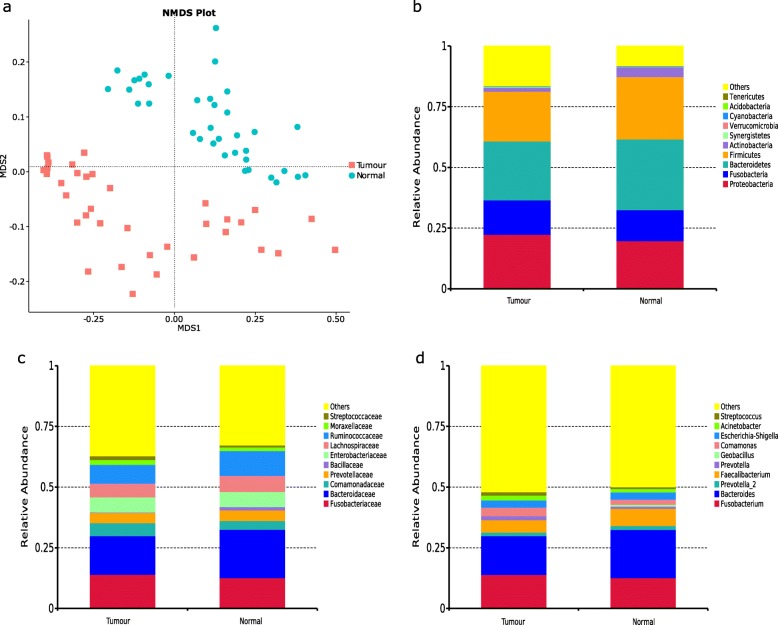
Microbial composition comparison between tumour tissues and paired normal tissues in CRC patients **a**. NMDS plot of microbiota based on unweighted UniFrac metric on OTUs. Each point represents one sample with colour indicating tumour tissues (red) and adjacent normal tissues (blue). **b-d**. Distribution of the top 10 most abundant taxa in the samples at phylum (**b**), family (**c**) and genus (**d**) level

Non-metric multidimensional scaling (NMDS) analysis based on the unweighted UniFrac distance on operational taxonomic units (OTUs) revealed that the microbial community composition of the cancerous tissues could be clearly distinguished from the non-cancerous tissues, which was confirmed by analysis of similarities (Anosim) (*p*-value = 0.002; Fig. 1a).

The microbial population structure differed between tumour and healthy tissues at multiple levels (Fig. 1b-d). At the phylum level, lower abundance of Bacteroidetes, Firmicutes and Actinobacteria, and higher abundance of Proteobacteria and Fusobacteria, was observed in tumour tissues (*p*-value > 0.05). At the family level, there was an under-representation of *Bacteroidaceae, Lachnospiraceae* and *Ruminococcaceae* and predominance of *Fusobacteriaceae* in tumour tissues (*p*-value > 0.05)*.* At the genus level, the most distinctive genera were *Fusobacterium, Bacteroides* and *Faecalibacterium*, with *Fusobacterium* higher in tumour tissues and the other two lower in tumour tissues (*p*-value > 0.05).

**Fig. 2:**
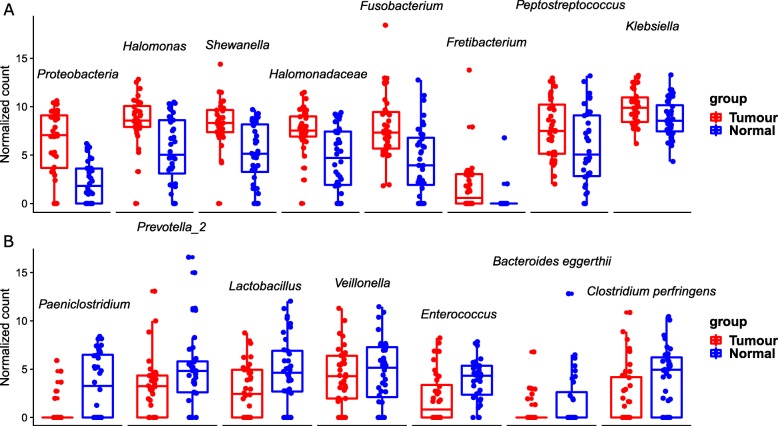
Boxplots of the differences in the mucosal microbiome between tumour tissues (red) and normal tissues (blue). Y axis: Relative counts normalized and log transformed by the default method in metagenomeSeq package. X axis: names of the significantly differentially abundant microbial taxa

To further characterise the bacterial genera driving the difference between the paired samples, the paired zero-inflated Gaussian (ZIG) mixture model was used [20]. As shown in Fig. 2, 15 taxa were identified to be the most relevant to the differences between tumour and normal tissues (Additional file 2: Table. S1). We found that taxa in family *Halomonadaceae,* genus *Halomonas,* genus *Shewanella,* genus *Fusobacterium,* genus *Fretibacterium,* genus *Peptostreptococcus* and genus *Klebsiella* were highly enriched in cancer tissues (*p*-value< 0.05, Fig. 2a; details in Additional file 2: Table. S1), while genus *Paeniclostridium,* species *Bacteroides eggerthii,* genus *Lactobacillus,* genus *Prevotella 2, Enterococcus,* species *Clostridium perfringens* and genus *Veillonella* were less abundant in cancer tissues (*p*-value < 0.05, Fig. 2b; Additional file 2: Table. S1).

Taken together, these results reveal the different microbial environments between the cancerous and normal colon mucosa.

### Microbiota associated with CRC-related colonic metabolites

**Fig. 3:**
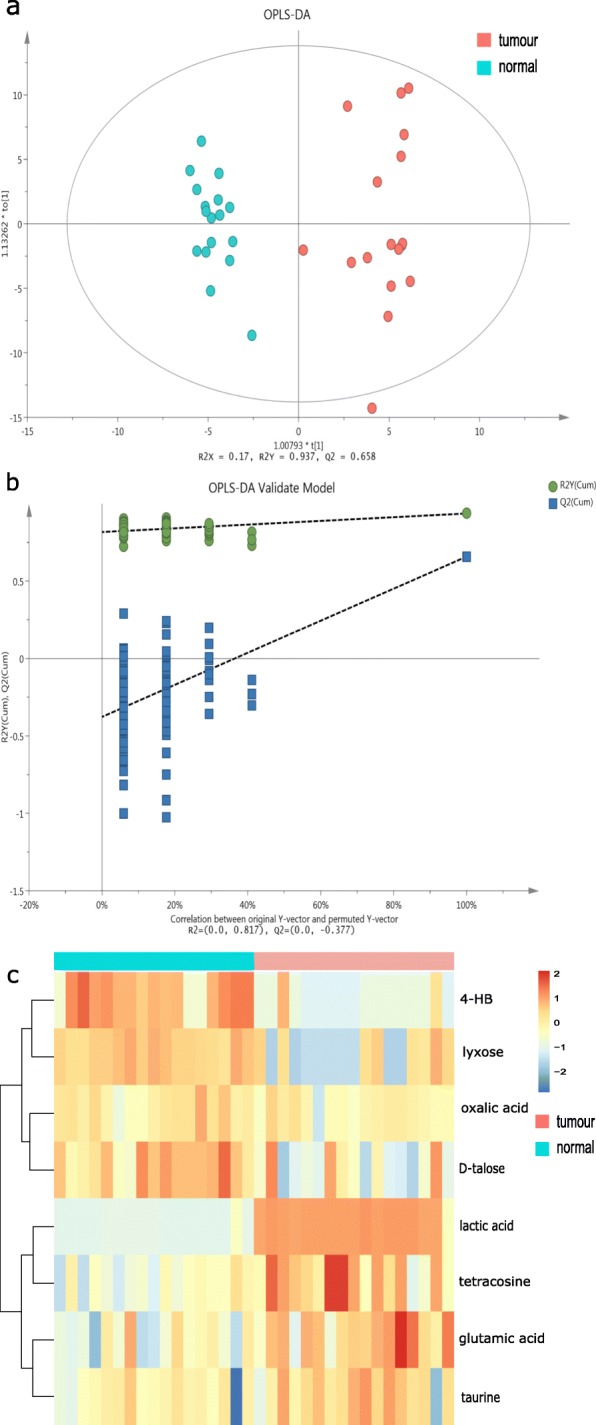
Comparison of the mucosal metabolome between tumour tissues and normal tissues in CRC patients **a**. OPLS-DA scores plot of metabolome between tissue samples. Each point represents one sample with colour indicating tumour tissues (red) and adjacent normal tissues (blue). **b**. OPLS-DA model validation based on seven-fold cross-validation and 200 permutation tests. Y axis: the R2 (green; goodness of fit) and Q2 (blue; predictability) of the all newly calculated and the original OPLS-DA models. X axis: correlation between the original Y observation and the permuted Y observation. R^2^_inter_ and Q^2^_inter_ are the intercept of the linear regression of R^2^ and Q^2^ from random models against those from original model. The negative Q^2^_inter_ indicated the validity of the model. **c**. Heatmap visualizing the significantly differentially abundant metabolites between tumour (red) and normal tissues (blue) based on hierarchical clustering analysis. The rows demonstrate the metabolites and the columns display samples

Seventeen pairs of samples were retained for colonic metabolome analysis, fifteen of which had matched microbiome data. In total, 309 metabolites were detected and quantified using the untargeted gas chromatography-mass spectrometry (GC-MS) approach. The score plot from orthogonal partial least squares discriminant analysis (OPLS-DA) revealed differences in metabolic features between the tissues (R^2^Y = 0.937; Q^2^ = 0.658; Fig. 3a). The reliability of the model was confirmed by seven-fold cross-validation and 200 response permutation tests on Fig. 3b, which has showed that all permutated values were lower than the original value. It indicated that the original classification model predicted the class better than the other newly calculated OPLS-DA models with randomly assigned class labels (Q^2^_inter_ = − 0.377; Fig. 3b).

Differential abundant metabolites were further identified based on Variable Influence on Projection (VIP) from the OPLS-DA model (threshold > 1) and significance level of 0.05 in the two-tailed paired t test. As shown in Fig. 3c, eight metabolites were selected, including lactic acid, 4-hydroxybutyric acid (4-HB), glutamic acid, taurine, lyxose, tetracosane, oxalic acid and D-talose. The heatmap revealed that the levels of 4-HB, lyxose, oxalic acid and D-talose were lower in tumour tissues compared to the paired normal tissues. On the other hand, the concentrations of lactic acid, glutamic acid, taurine and tetracosane were elevated in tumour tissues.

Pearson correlation analysis between changes in the level of the differentially abundant metabolites and the microbial genera in cancer and healthy tissues revealed several significant associations. The elevated abundance of genus *Fusobacterium* was significantly correlated with the decreased 4-HB level (*r* = 0.722, *p*-value = 0.002, degree of freedom(df) = 13; Table 1; details in Additional file 3: Figure S2). On the other hand, decrease in abundance of genus *Prevotella 2* was correlated with the declined level of 4-HB in tumour tissues in comparison with matched normal tissues (*r* = − 0.593, *p*-value =0.02, df = 13; Table 1). In addition, the increase of family *Halomonadaceae* abundance exhibited a significant correlation with the increase in glutamic acid level between the tissues (*r* = 0.541, *p*-value = 0.037, df = 13; Table 1). The increase of lactic acid concentration in cancer tissues was also found to be significantly associated with the decrease in both genus *Paeniclostridium* level (*r* = − 0.537, *p*-value = 0.039, df = 13; Table 1) and genus *Enterococcus* level (*r* = − 0.527, *p*-value = 0.044, df = 13; Table 1).

**Table 1:** Significant correlation of differentially abundant microbial taxa and metabolites (*n* = 15)

Microbial taxa	Metabolites	r value^a^	*p*-value
Genus *Fusobacterium* (increased in tumour)	4-HB (decreased in tumour)	0.722	0.002
Genus *Prevotella 2* (decreased in tumour)	4-HB (decreased in tumour)	−0.593	0.020
Family *Halomonadaceae* (increased in tumour)	glutamic acid (increased in tumour)	0.541	0.037
Genus *Paeniclostridium* (decreased in tumour)	lactic acid (increased in tumour)	−0.537	0.039
Genus *Enterococcus* (decreased in tumour)	lactic acid (increased in tumour)	−0.527	0.044

^a^For the calculation of the correlations, the signed relative change of microbe abundance and metabolite level between paired tumour and normal tissues were used

### Colonic metabolite-correlated microbial taxa associated with DNA methylation-related differential gene expression

**Fig. 4:**
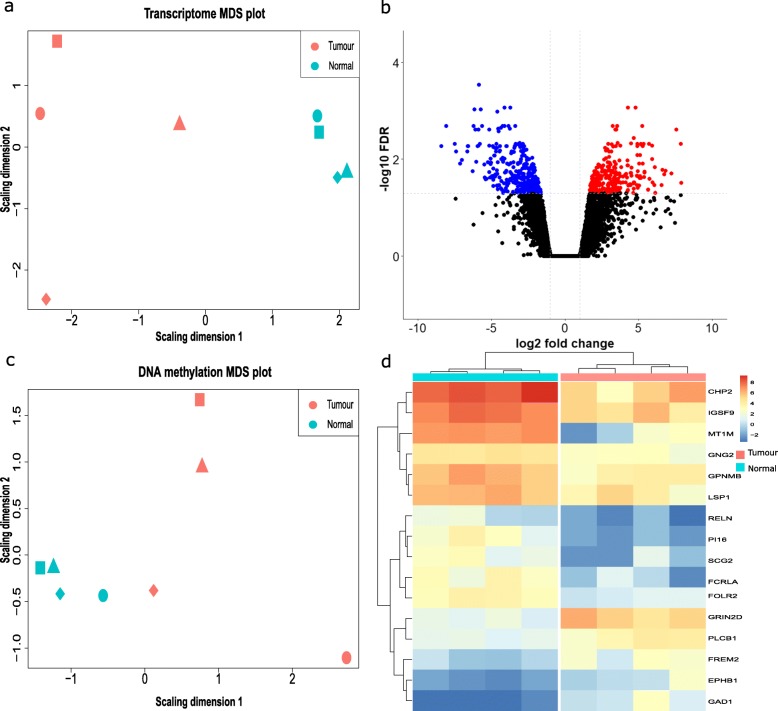
Comparison of the mucosal transcriptome and DNA methylation profile between tumour tissues and normal tissues **a**. MDS plot of transcriptome between tissue samples. Each point represents one sample with colour indicating tumour tissues (red) and adjacent normal tissues (blue), shape indicating the patient. **b**. Volcano plot of the significant differences in the mucosal transcriptome between paired samples. The x-axis shows the gene expression log_2_FC between paired samples; the y-axis represents minus log_10_(FDR-adjusted *p*-value) calculated from GLM. Genes with more than two fold change in expression level are coloured in red (upregulated in tumour tissues) and green (downregulated in tumour tissues). **c**. MDS plot of DNA methylation for differentially expressed genes between paired tissue samples. Each point represents one sample with colour indicating tumour tissues (red) and adjacent normal tissues (blue), shape indicating the patient. **d**. Heatmap visualizing the DNA methylation related differentially expressed genes between tumour (red) and normal tissues (blue) based on hierarchical clustering analysis. The rows demonstrate the genes and the columns display samples

In total, four tumour and normal tissue pairs were selected for transcriptome analysis using RNA-seq. After pre-processing and filtering, 15,832 gene transcripts were included in downstream analysis. Multi-dimensional scaling (MDS) was performed to visualise the differences in gene expression levels between cancer tissues and matched healthy tissues (Fig. 4a). After generalized linear model (GLM)-based paired differential comparison, 261 transcripts were found to be down-regulated in cancer tissues while 333 were found to be significantly up-regulated in tumour tissues (fold change (FC) > 2, *p*-value< 0.05; Fig. 4b).

We then evaluated the methylation profile of four tumour and normal tissues pairs at the 594 differentially expressed genes, including all the probes that cover the promoters and gene body of these genes. After quality control, 14,108 probes were selected for subsequent analysis. As shown in Fig. 4c, the methylation patterns of these differentially expressed genes are clearly distinctive between the tumour and normal tissues. Of the 111 differentially methylated probes, 68 were highly methylated in cancer tissues (*p*-value< 0.05; Additional file 4: Table. S2). Due to the reported relationship of DNA hypermethylation located in gene promotor and gene transcription repression, genes with the opposite trend of DNA methylation at the promoter region and the corresponding gene expression level were considered as DNA methylation-related differentially expressed genes. This included sixteen genes: *CHP2*, *EPHB1*, *FCRLA*, *FOLR2*, *FREM2*, *GAD1*, *GNG2*, *GPNMB*, *GRIN2D*, *IGSF9*, *LSP1*, *MT1M*, *PI16*, *PLCB1*, *RELN* and *SCG2* (Fig. 4d).

We further validated our hypothesis of a possible microbiome and host transcriptome interaction by analysing the correlation between the two profiles. The microbial taxa that were associated with the colonic metabolome were included. As a result, the increase in genus *Fusobacterium* abundance was found to be significantly associated with the down-regulated expression of *PI16* (*r* = − 0.993, *p*-value = 0.008, df = 2; Table 2) and also significantly associated with the decreased expression of *FCRLA* (*r* = 0.956, *p*-value = 0.044, df = 2; Table 2) and *LSP1* (*r* = 0.954, *p*-value = 0.046, df = 2; Table 2) in cancer tissues in comparison with the healthy tissues. The decrease in the abundance of genus *Prevotella 2* was significantly associated with the decreased expression of *MT1M* in cancer tissues (*r* = − 0.999, *p*-value = 0.001, df = 2; Table 2); the positive abundance change of family *Halomonadaceae* was significantly associated with the reduction in the expression level of *RELN* (*r* = 0.957, *p*-value = 0.043, df = 2; Table 2). Nevertheless, the reduced abundance of genus *Paeniclostridium* and genus *Enterococcus* in cancer tissues also exhibited significant associations with the expression difference of *PLCB1* (*r* = 0.995, *p*-value = 0.01, df = 2; Table 2) and *IGSF9*, respectively (*r* = − 0.997, *p*-value = 0.003, df = 2; Table 2).

**Table 2:** Significant correlation of colonic metabolite-correlated microbial taxa and DNA methylation-related differential gene expression (*n* = 4)

Microbial taxa	Genes	Coding protein	r value^a^	*p*-value
Genus *Fusobacterium* (increased in tumour)	*PI16* (decreased in tumour)	Peptidase inhibitor 16	−0.993	0.008
	*FCRLA* (decreased in tumour)	Fc Receptor Like A	0.956	0.044
	*LSP1* (decreased in tumour)	Lymphocyte Specific Protein 1	0.954	0.046
Genus *Prevotella 2* (increased in tumour)	*MT1M* (decreased in tumour)	Metallothionein	−0.999	0.001
Family *Halomonadaceae* (increased in tumour)	*RELN* (decreased in tumour)	Reelin	0.957	0.043
Genus *Paeniclostridium* (decreased in tumour)	*PLCB1* (increased in tumour)	Phospholipase C beta 1	0.995	0.010
Genus *Enterococcus* (decreased in tumour)	*IGSF9* (decreased in tumour)	Immunoglobulin Superfamily Member 9	−0.997	0.003

^a^For the calculation of the correlations, the signed relative change of microbe abundance and gene expression level between paired tumour and normal tissues were used

## Discussion

In our present study, we comprehensively investigated the host-microbiome interaction in CRC by assessing multi-omics, including the mucosal microbiome, mucosal metabolome, host transcriptome and host DNA methylation profile.

We replicated several differentially abundant microorganisms as reported in previous CRC-related studies. At the genus level, potential pathogenic microorganisms such as genus *Fusobacterium* were found to be significantly enriched in tumour tissues compared with normal tissues in our samples. The genus *Fusobacterium* is a well-known potential pathogenic gut microorganism and enrichment of this genus has been reported to be associated with CRC in several studies [21–23]. The over-representation of genus *Halomonas* and genus *Shewanella* has also been revealed in tumour-associated microbiota in patients with rectal and distal colon cancers [24]. In addition, genus *Peptostreptococcus* has been shown to be associated with CRC in several studies [8, 25, 26]. Similarly, genus *Klebsiella* has been implicated in the progression of CRC [6, 27]. On the other hand, genus *Lactobacillus* and genus *Enterococcus,* were over-represented in healthy tissues in the current study, which have been long regarded as anti-inflammation and anti-tumorigenic probiotics [28, 29]. A recent study also reported that genus *Lactobacillus* was able to counterbalance the dysbiosis induced by CRC in human and mice [26, 30]. In an animal study, genus *Enterococcus* was shown to inhibit chemically-induced CRC [31].

The microbiota and its metabolic products have been reported to affect the health status of the host, especially in colon tissues [32]. In line with other metabolome studies in CRC, several metabolites were found to be significantly differentially abundant in cancer mucosal tissues in contrast to adjacent healthy tissues. Interestingly, the top three most distinguished differences between colon tissues in our study were lactic acid, 4-hydroxybutyric acid and glutamic acid. The elevation of lactic acid and glutamic acid in cancer tissues has been shown in multiple cohort studies of the CRC mucosa metabolome [33]. Additionally, taurine was found to be high in tumour tissues. Taurine is a sulphur amino acid, and studies have concluded that it is pro-inflammatory and genotoxic [34]. Oxalic acid has also been reported to be decreased in CRC samples [35]. Though tetracosane has been reported to be cytotoxic and potentially apoptotic, a high level of tetracosane in CRC tumour tissues has not yet been reported and warrants further study [36]. D-talose, which was found to be more abundant in normal tissues, is a rare sugar with possible anti-tumour application in the pharmaceutical industry [37, 38].

The key findings in our study were the associations between changes in microbial taxa, butyrate-related colonic metabolites, and DNA methylation-associated host gene expression between tumour and paired normal colon tissues. We identified a significant association between the increase of the pro-oncogenic genus *Fusobacterium* and decrease of 4-HB between the tissues. As a hydroxylated derivative of butyrate, 4-HB is involved in one of the four pathways of butyrate synthesis (the 4-aminobutyrate pathway) [39]. Although genus *Fusobacterium*, especially *F. nucleatum,* has been linked to colorectal tumorigenesis, the mechanism remains unclear [21–23]. Our finding suggests that, under tumour conditions, genus *Fusobacterium* might promote inflammatory response and carcinogenesis by interfering with butyrate synthesis in the tumour tissues. Butyrate is a short-chain fatty acid and has been revealed in several previous studies to be a potential anti-carcinogenic and anti-inflammatory metabolite [40, 41].

Butyrate is produced by the microbial fermentation of undigested polysaccharides that reach the colon [42]. In addition, butyrate also plays a crucial role in influencing gene expression in host colonic cells via epigenetic regulation [43, 44]. In human cells, there is evidence that butyrate has a direct effect on DNA methylation by regulating key enzymes, including methylcytosine dioxygenase (TET) and DNA methyltransferase 1 (DNMT1) [45, 46]. Butyrate has also been shown to modify DNA methylation and inhibit Wnt signalling in human gastric cancer cells, a pathway also known to be activated in CRC [47]. The potential association between *Fusobacterium* and DNA methylation-related carcinogenesis has been supported by a previous study [18]. It has also been demonstrated that *Fusobacterium* is enriched in CRC tissues, especially those with the methylation phenotype [22]. Butyrate may mediate the effect of *Fusobacterium* on host DNA methylation modifications.

In our study, the change in abundance of genus *Fusobacterium* was also correlated with DNA methylation-related expression differences in genes *PI16*, *FCRLA* and *LSP1* in colon tissues. *PI16* is a peptidase inhibitor; consistent with our findings, other studies have detected decreased expression of *PI16* in colon cancer [48]. *FCRLA* is an unusual member of the lymphocyte receptor family and is associated with multiple Ig isotypes, including IgM, IgG and IgA [49, 50]. As a B cell-specific protein, *FCRLA* may play a crucial role in the mucosal immune response [51]. Down-regulation of *LSP1* has been shown in several cancers and its over-expression is associated with cell apoptosis [52, 53].

Additionally, butyrate might also play a role in the effect of genus *Prevotella 2* on CRC. The decrease of genus *Prevotella 2* abundance and 4-HB concentration in tumour tissues showed a significant correlation. In terms of the correlation between genus *Prevotella 2* and 4-HB, more detailed genomic analysis of its strains is warranted to identify the metabolic pathway. The change of genus *Prevotella 2* abundance was also associated with the reduced expression of *MT1M* in cancer tissues compared with healthy tissues. A member of the metallothionein (MT) family, *MT1M* is a cysteine-rich metal-binding protein that is induced by inflammation and protects the cell against carcinogens [54].

Another observation in our study is the positive relationship between the increase of glutamic acid and microbial taxa in the potential pathogenic family *Halomonadaceae* in cancer tissues compared with normal tissues*.* In the large intestine, glutamic acid is partially originated from the digestion of alimentary and endogenous proteins by the colonic microbes [55]. Glutamic acid can also serve as butyrate precursor and at least five various pathways have been reported to be responsible for the fermentation of glutamic to butyrate by anaerobic bacteria [56]. Nonetheless, glutamic acid can also be metabolized to α-ketoglutarate (α-KG), which is co-factor of methylcytosine dioxygenase (TET) [57]. The link between *Halomonadaceae* and glutamic acid identified in our finding suggests that taxa in this family might involve in glutamic acid metabolism. Furthermore, the elevation of the taxa in family *Halomonadaceae* also correlated with the decreased expression of Reelin (*RELN*) in tumour tissues. The downregulation of Reelin has been found to be related to cell migration ability, tumour invasiveness and patients’ survival rates in several cancers, including gastric, breast, pancreatic and liver cancers [58–60]. Importantly, large CpG islands are located at Reelin promoter sites and its transcriptional silence has been shown to be strongly controlled by promoter hypermethylation [61]. Our finding might offer additional evidence of the mechanism of epigenetic regulation of *RELN* in colon cancer.

We also reported a significant association between the increase in lactic acid level and the decrease in abundance of genus *Paeniclostridium* as well as genus *Enterococcus* in cancer tissues, in contrast with normal tissues. This may in part be due to the excess lactic acid produced by cancer cells as a result of the high consumption of glucose in glycolysis, which is characteristic of tumour metabolism; this is termed the Warburg Effect [62]. Nonetheless, due to the synergic metabolism of lactate-producers such as genus *Enterococcus* and lactate-utilizing bacteria in the colon, less lactate might be disposed by lactate-utilizing bacteria in tumour tissues [63]. Lactate fermented by lactate-utilizing bacteria can be used to generate butyrate, which, as mentioned, might influence gene expression by modifying DNA methylation [64]. To explain the correlation between genus *Paeniclostridium* and lactic acid, further functional studies are required to explore their relationship. Interestingly, the reduced level of genus *Enterococcus* in tumour tissues was also found to be associated with the down-regulated expression of *IGSF9* (Immunoglobulin Superfamily Member 9), which contributes to regulating immune cell activation [65]. Moreover, the reduced abundance of genus *Paeniclostridium* was related to the increased expression of Phospholipase C beta 1 (*PLCB1*). Phospholipase C (PLC) is critical for cell proliferation and apoptosis in several cell systems [66, 67]. The knockdown of *PLCB1* is reported to initiate apoptosis, leading to strong growth arrest [68].

One limitation of our study is the use of 16sRNA sequencing technology; as such, we are unsure which specific species might distinguish tumour and normal tissues. However, the current shotgun metagenomic method is infeasible for mucosal microbiome study because of human DNA contamination [69]. Future development of sequencing technology would be critical for the identification of CRC-related microbes. Due to a relatively small sample size, the heterogeneity of CRC was also not investigated. However, our main aim was to conduct a pilot study to investigate the potential interaction between the host and microbiome from multiple “omics”. Future studies with larger sample sizes would help disentangle the complicated host-microbiome relationship in different stages and different types of CRC. Another issue is the causality of the reported relationship. Further animal intervention studies are needed to validate the correlation identified from our findings.

Apart from integrating multiple “omics”, another strength of our study is that we measured the microbiome from the mucosa sampled from CRC patients. The mucosal microbiome is different from the faecal microbiome, which is only an estimate of the former. The mucosal microbiome exhibits a more intimate and direct interaction with the host colonic epithelium; thus, it has a greater impact on the pathogenesis of disease [70].

## Conclusions

In conclusion, our study identified associations between previously reported CRC-related microbial taxa, butyrate-related metabolties and DNA methylation-associated gene expression in tumour and paired normal colonic mucosa tissues from CRC patients, which uncovered a possible mechanism of the role of microbiome in the carcinogenesis of CRC. In addition, these findings offer insights into potential new biomarkers, therapeutic and/or prevention strategies for CRC.

## Methods

### Patient recruitment and DNA/RNA isolation

A total of 36 pairs of tumour tissues and adjacent healthy colon tissues were obtained from CRC patients in the Department of Colorectal and Anal Surgery, First Affiliated Hospital of Zhejiang University, China. Mucosa tissues were collected during surgery from both the tumour site and from an adjacent non-tumorous site that was more than 5 cm away from the tumour. The samples were flash-frozen in liquid nitrogen immediately after colonic resection and were stored at − 80 °C. Patients with diabetes or infectious diseases, those undergoing radiotherapy or chemotherapy, and those who had received antibiotics or probiotics within 4 weeks were excluded from the study. The study was approved by the Ethics Committee of the First Affiliated Hospital of the Medical School of Zhejiang University. Written informed consent was obtained from all subjects.

Total DNA and RNA were extracted from the tissue samples using AllPrep DNA/RNA mini kit (Qiagen, Valencia, CA, USA) according to the manufacturer’s instructions.

### Mucosal microbiome analysis

After assessing DNA concentration and quality, PCR amplification of the V3-V4 regions of the 16S rRNA gene was performed. Then, the qualified amplicon libraries were sequenced on the Ion S5TM XL platform (Thermo Scientific) following the manufacturer’s protocol. The sequencing reads were processed using QIIME pipeline (v1.9.1) [71]. Quality filtering were performed under specific conditions according to the Cutadapt (Martin, 2011) [72]. Chimera sequences were identified using the UCHIME algorithm and were removed. After quality control and trimming, the reads were merged and clustered into operational taxonomic units (OTUs) using UPARSE software (v7.0.1001) with a 97% similarity threshold [73]. Taxonomy was assigned to each OTU cluster based on the representative reads using the RDP classifier based on the SILVA Database version 123 (confidence threshold: 80%) [74]. OTUs present in less than 25% of the total samples and with less than 3 reads were excluded. Non-metric multidimensional scaling (NMDS) was performed to visualise between-sample diversity based on unweighted UniFrac distances on OTUs using the *vegan* package with R software (v 2.15.3). Additionally, Analysis of similarities (Anosim) was conducted to statistically test the significance of the separation between samples. The default cumulative sum scaling (CSS) normalization procedure in *metagenomeSeq* R package and log transformation was used to normalize microbiome data. The paired zero-inflated Gaussian (ZIG) mixture model from the *metagenomeSeq* R package was applied to identify statistically significant and biologically relevant taxa in paired cancer and healthy colon mucosa samples [20].

### Mucosal metabolomics analysis

50 mg of colon mucosa tissues was mixed with 800 μl ice-cold methanol and homogenized. After centrifugation, the supernatant was transferred to a new Eppendorf tube containing 20 μl of internal standard (1 mg/ml heptadecanoic). The remainder was extracted with 800 ul ice-cold methanol/chloroform (3:1, v/v) and the supernatant was then combined with the supernatant from the previous step. Next, the samples were dried under a nitrogen stream (Aosheng, Hangzhou, China). The dried residue was added to 80 μl of 20 mg/ml methoxylamine hydrochloride in anhydrous pyridine. The mixture was incubated at 37 °C for 24 h after vortexing. Then, the sample was mixed with 100 μl of N,O-bistrifluoroacetamide (BSTFA) [with 1% trimethylsilyl chloride (TMCS)] (Sigma-Aldrich, St. Louis, MO, USA) and derivatised (70 °C for 2 h). Untargeted metabolomics analysis was carried out by gas chromatography-mass spectrometry (GC-MS) with an Agilent 7890A GC and 5975C inert mass selective detector (MSD) system (Agilent Technologies, Santa Clara, CA, USA). The raw data from GC-MS was transformed by ChemStation software (version E.02.02.1431, Agilent, CA, USA) and pre-treated by ChromaTOF software (version 4.34, LECO, St. Joseph, MI, USA). The Fiehn database was referred to for identification of the metabolites. The GC-MS dataset was quantile normalized, auto-scaled and log transformed using MetaboAnalyst 4.0 (www.metaboanalyst.ca/). Subsequently, principal component analysis (PCA) and orthogonal partial least squares discriminant analysis (OPLS-DA) was performed using SIMCA (Umetrics, Umeå Sweden) to measure the differences in metabolites between the tissue samples. Model quality was summarized by R2Y and Q2 parameters, which measures the goodness of fit and the predictive ability of the model respectively (26). To measure the reliability of the OPLS-DA model from overfitting, we performed seven-fold cross-validation and 200 response permutation tests which randomly permutated the group attributes (Y) whereas the variables (X) remained same. Metabolites were considered statistically significant if the Variable Influence on Projection (VIP) value, a measure of the relative influence on the model, was greater than 1 and the *p*-value returned from the two-tailed Student’s t test on normalized data was less than 0.05.

### Host transcriptome analysis

The RNA sequencing library was prepared as previously described [75] and sequenced on an Illumina Hiseq 2000 platform. After quality control, the paired-end clean reads were mapped to the reference genome hg19 using Hisat2 (v2.0.5). FeatureCounts (v1.5.0-p3) was used to count the number of reads mapped to genes [76]. Then, the gene expression value was quantified by the length of the gene and total read counts of the gene as count per million (CPM). The transcripts were subsequently filtered with the cut-off of CPM > 0.5 in at least 4 libraries. The bioconductor package *edgeR* was used to normalize and analyse differential gene expression levels between paired cancerous and non-cancerous tissues with a generalized linear model (GLM) [77]. Multi-dimensional scaling (MDS) plot was generated to visualize the difference between pairs of RNA samples. The resulting *p-*values were corrected using the Benjamini and Hochberg approach at False Discovery Rate (FDR) of 5% [78]. Genes with an adjusted *p-*value< 0.05 and fold change (FC) > 2 were considered differentially expressed between the groups.

### Host methylation analysis

An EZ DNA Methylation Kit was used for bisulfite modification of the genomic DNA. Then, DNA methylation analysis was performed using the Infinium HumanMethylation850 BeadChip array (Illumina San Diego, CA, USA), which covers more than 850,000 CpG sites and 99% of the RefSeq genes. DNA methylation data analysis was performed following a cross-package Bioconductor workflow [79]. Methylation levels were quantified as beta value, which describes the proportion of methylation at each CpG locus. After background subtraction and data normalization, probes that were located at the region of the differentially expressed genes were selected for subsequent analysis. Differential DNA methylation between the groups was assessed by paired linear model using the *limma* R package and the *p*-values were adjusted for multiple testing using Benjamini-Hochberg method at FDR of 5% [78]. The probes with an adjusted *p*-value less than 0.05 were considered statistically significantly differentially methylated.

### Interactions between omics

The correlations between change in the level of differentially abundant microbes and metabolites were estimated with Pearson correlation tests using the function cor.test in R (version 3.5.2). Due to the interdependence between paired tumour and normal tissues, the signed relative change of microbe abundance and metabolite concentration between paired tissues were used for the calculation of the correlations. Correlations were considered statistically significant if the *p-*value was less than 0.05. The same correlation tests were also conducted between the levels of differentially abundant microbes and differentially expressed genes that also exhibited opposite trend of differential methylation at the promoter region. Metabolites and methylation-related differentially expressed genes that were associated with the same microbe were reported.

## Supplementary information

**Additional file 1 Figure S1.** Flow chart of study design.

**Additional file 2 Table S1.** Significantly differentially abundant microbial genera between tumour and normal colon tissues from CRC patients (*n* = 36).

**Additional file 3 Figure S2.** Illustration of correlation between microbial abundance and metabolite concentration.

**Additional file 4 Table S2.** Significantly differentially methylated probes that located within the differentially expressed genes between tumour and normal colon tissues from CRC patients (*n* = 4).

## Data Availability

The datasets used and/or analysed during the current study are available from Sequence Read Archive (SRA) database (accession number: PRJNA599023). All data generated or analysed during this study are also available from the corresponding author on reasonable request.

## Abbreviations

CRC: Colorectal cancer
NMDS: Non-metric multidimensional scaling
OTU: Operational taxonomic unit
Anosim: Analysis of similarities
FDR: False discovery rate
ZIG: Zero-inflated Gaussian
GC-MS: Gas chromatography-mass spectrometry
PCA: Principal component analysis
OPLS-DA: Orthogonal partial least squares discriminant analysis
VIP: Variable Influence on Projection
MDS: Multi-dimensional scaling
GLM: Generalized linear model
FC: Fold change
4-HB: 4-hydroxybutyric acid
DNMT1: DNA methyltransferase 1
TET: Methylcytosine dioxygenase
