# Supplementary material for: Multi-omic profiling reveals associations between the gut mucosal microbiome, the metabolome, and host DNA methylation associated gene expression in patients with colorectal cancer
Source: BMC Microbiol. 2020 Apr 23;20(Suppl 1):83. doi: 10.1186/s12866-020-01762-2 (PMC7178946; doi:10.1186/s12866-020-01762-2)
Supplement: Supplementary file 1 — Additional file 1 Figure S1. Flow chart of study design. [file 12866_2020_1762_MOESM1_ESM.docx]

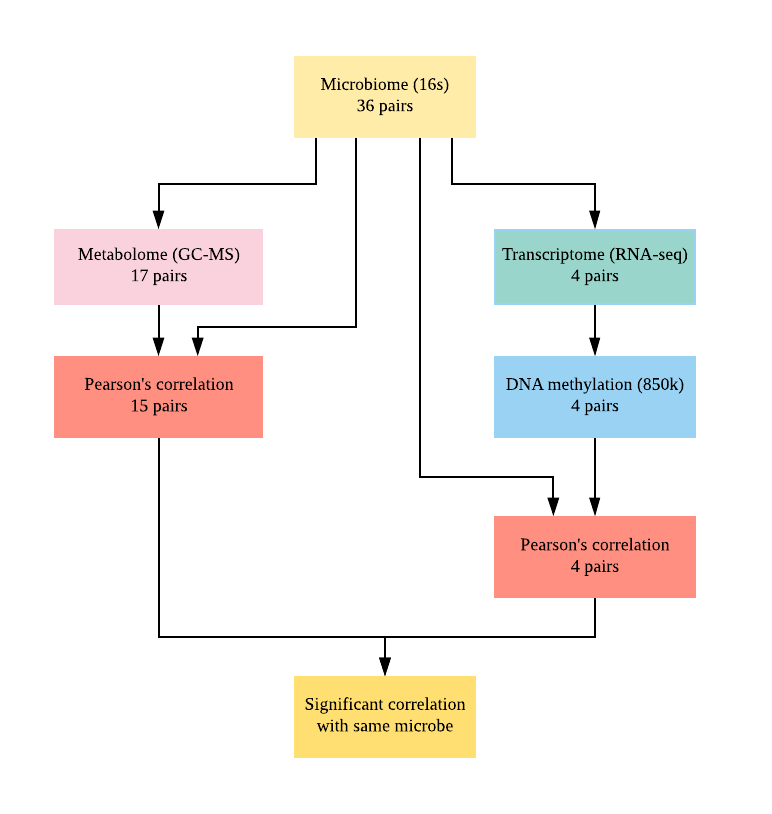
 Figure S1. Flow chart of study design

This is a brief overview of the design of this study. First, a total of thirty-six matched tumour and normal pairs were collected for microbiome analysis using 16S rRNA sequencing. Then, a total of seventeen pairs of the matched colon tissues were retained for metabolome analysis using the untargeted GC-MS approach. In addition, four matched colon tissue pairs were used for transcriptome analysis (RNA-seq) and for DNA methylation analysis (Infinium HumanMethylation850 BeadChip array). Next, Pearson correlation analysis between differentially abundant microbes and metabolites were conducted on fifteen pairs of tissues on which both datasets were available. Similarly, the Pearson correlation between differentially abundant microbes and DNA methylation-related differentially expressed genes were calculated for four pairs of match tissues that have all the mentioned datasets. Finally, differentially expressed genes and differentially abundant metabolites that were correlated with the same microbial taxa were reported.
