# Supplementary material for: Multi-omic profiling reveals associations between the gut mucosal microbiome, the metabolome, and host DNA methylation associated gene expression in patients with colorectal cancer
Source: BMC Microbiol. 2020 Apr 23;20(Suppl 1):83. doi: 10.1186/s12866-020-01762-2 (PMC7178946; doi:10.1186/s12866-020-01762-2)
Supplement: Supplementary file 2 — Additional file 2 Table S1. Significantly differentially abundant microbial genera between tumour and normal colon tissues from CRC patients (n = 36). [file 12866_2020_1762_MOESM2_ESM.docx]

**Table S1 Significantly differentially abundant microbial genera between tumour and normal colon tissues from CRC patients (*n*=36).**

| **Microbial taxa** | **statistic** | **p-value** | **adjp-value** |
| --- | --- | --- | --- |
| *Proteobacteria* | -3.83833 | 1.61 × 10^-18^ | 1.65 × 10^-16^ |
| *Halomonas* | -3.24738 | 6.51 × 10^-19^ | 1.01 × 10^-16^ |
| *Shewanella* | -3.00474 | 6.26 × 10^-16^ | 4.83 × 10^-14^ |
| *Halomonadaceae* | -2.90673 | 1.79 × 10^-14^ | 1.11 × 10^-12^ |
| *Fusobacterium* | -2.21731 | 2.31 × 10^-9^ | 1.19 × 10^-7^ |
| *Fretibacterium* | -1.99084 | 0.000594 | 0.016368 |
| *Peptostreptococcus* | -1.25626 | 0.000636 | 0.016368 |
| *Klebsiella* | -1.08806 | 0.00207 | 0.041641 |
| *Veillonella* | 1.396755 | 0.000374 | 0.011554 |
| *Clostridium perfringens* | 1.421805 | 0.002156 | 0.041641 |
| *Enterococcus* | 1.438088 | 0.001197 | 0.028462 |
| *Prevotella 2* | 1.736653 | 3.55 × 10^-5^ | 0.00137 |
| *Lactobacillus* | 1.891827 | 4.03 × 10^-5^ | 0.001383 |
| *Bacteroides eggerthii* | 1.909034 | 0.002131 | 0.041641 |
| *Paeniclostridium* | 2.882027 | 1.50 × 10^-6^ | 6.60 × 10^-5^ |
