# Supplementary material for: Multi-omic profiling reveals associations between the gut mucosal microbiome, the metabolome, and host DNA methylation associated gene expression in patients with colorectal cancer
Source: BMC Microbiol. 2020 Apr 23;20(Suppl 1):83. doi: 10.1186/s12866-020-01762-2 (PMC7178946; doi:10.1186/s12866-020-01762-2)
Supplement: Supplementary file 3 — Additional file 3 Figure S2. Illustration of correlation between microbial abundance and metabolite concentration. [file 12866_2020_1762_MOESM3_ESM.docx]

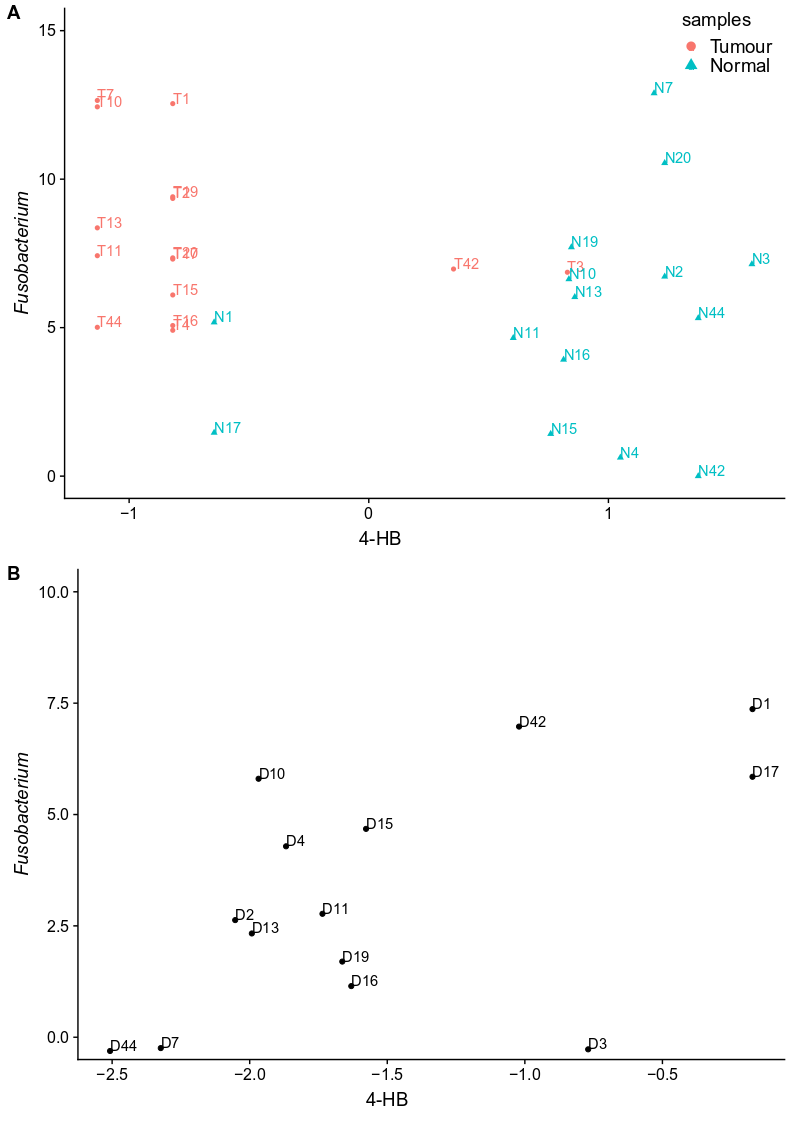


Figure S2.A Scatter plot of abundance of 4-hydrobutyrate(4-HB) and genus *Fusobacterium* in tumour and normal tissues. The x axis shows the quantile normalized, auto-scaled and log transformed abundance of 4-HB in tumour and normal tissues. The y-axis shows CSS normalized and log transformed abundance of genus *Fusobacterium* in tumour and normal tissues.

Figure S2.B Scatter plot of the abundance difference of 4-HB and genus *Fusobacterium* between tumour and paired normal tissues. The x axis shows 4-HB abundance difference (tumour-normal), the minus value means the level of 4-HB was lower in tumour tissue compared to normal tissue. The y axis shows genus *Fusobacterium* abundance difference (tumour-normal). Similarly, the positive value means the level of genus *Fusobacterium* was higher in tumour tissue compared to normal tissue. Thus, the plot displayed that the elevated abundance of genus *Fusobacterium* was accompanied by decreased 4-HB level in tumour tissue compared to the normal tissues.

With the example of 4-HB and genus *Fusobacterium,* supplementary Figure 2 described details about the correlation analysis in this paper. Due to the interdependence between paired tumour and normal tissue, the relative change of each omic (microbiome and metabolome, microbiome and gene expression) between paired tissues were used for the calculation of Pearson correlation. Thus, the correlation coefficients shown in the result (Table 1 and Table 2) described the relationship between the differences (increase or decrease) of microbial taxa abundance and metabolite concentration/DNA methylation-associated gene expression between tumour and normal tissues.
