# Supplementary material for: Multi-omic profiling reveals associations between the gut mucosal microbiome, the metabolome, and host DNA methylation associated gene expression in patients with colorectal cancer
Source: BMC Microbiol. 2020 Apr 23;20(Suppl 1):83. doi: 10.1186/s12866-020-01762-2 (PMC7178946; doi:10.1186/s12866-020-01762-2)
Supplement: Supplementary file 4 — Additional file 4 Table S2. Significantly differentially methylated probes that located within the differentially expressed genes between tumour and normal colon tissues from CRC patients (n = 4). [file 12866_2020_1762_MOESM4_ESM.docx]

**Table S2 Significantly differentially methylated probes that located within the differentially expressed genes between tumour and normal colon tissues from CRC patients (*n*=4).**

| **Symbol** | **probe** | **logFC** | **p-value** | **adjp-value** |
| --- | --- | --- | --- | --- |
| ANKRD13B | cg21468728 | 3.022536 | 0.000127 | 0.045957 |
| ARHGAP20 | cg21406100 | -4.31607 | 9.13 × 10^-6^ | 0.039106 |
| ARHGAP20 | cg24575245 | -6.02042 | 4.41 × 10^-5^ | 0.039106 |
| ARHGAP20 | cg12767055 | -4.13245 | 4.44 × 10^-5^ | 0.039106 |
| ASCL2 | cg10526374 | 2.023328 | 9.41 × 10^-5^ | 0.045957 |
| ASCL2 | cg22346124 | 2.538751 | 0.000103 | 0.045957 |
| ASCL2 | cg06272038 | 2.210216 | 0.000165 | 0.045957 |
| ASCL2 | cg05309948 | 1.971331 | 0.000226 | 0.046198 |
| ASCL2 | cg15790820 | 2.703258 | 0.000231 | 0.046198 |
| ASCL2 | cg21365566 | 2.205357 | 0.000348 | 0.049139 |
| C3 | cg05103863 | 2.119851 | 0.000157 | 0.045957 |
| C3 | cg17185886 | 2.351568 | 0.000369 | 0.049139 |
| CAMK2N1 | cg02034779 | -1.90252 | 0.000218 | 0.046198 |
| CCDC68 | cg26060179 | -1.59857 | 0.000254 | 0.046198 |
| CHP2 | cg08404028 | 2.469086 | 0.000217 | 0.046198 |
| CHST4 | cg00840403 | 3.957303 | 0.000139 | 0.045957 |
| CHST4 | cg02954149 | 3.249734 | 0.000312 | 0.047277 |
| COLEC12 | cg07137336 | -3.65973 | 0.000319 | 0.047859 |
| CPNE8 | cg26510017 | -3.74381 | 0.000356 | 0.049139 |
| CPNE8 | cg13509195 | -3.56575 | 0.000382 | 0.049336 |
| CRNDE | cg01686920 | 1.7808 | 0.000278 | 0.046198 |
| EPHB1 | cg10608570 | -3.87509 | 2.28 × 10^-5^ | 0.039106 |
| FABP6 | cg01711005 | 1.970841 | 0.000185 | 0.046198 |
| FCRLA | cg05033369 | 2.446298 | 0.000167 | 0.045957 |
| FOLR2 | cg03919729 | 2.117458 | 0.000354 | 0.049139 |
| FOXA2 | cg07003030 | 1.596127 | 0.000274 | 0.046198 |
| FREM2 | cg16740299 | 2.175557 | 0.000229 | 0.046198 |
| FREM2 | cg26063106 | -1.5045 | 0.000267 | 0.046198 |
| GAD1 | cg15753746 | -2.25813 | 0.000138 | 0.045957 |
| GAD1 | cg25264004 | -4.01103 | 0.000148 | 0.045957 |
| GAD1 | cg11266473 | -1.92723 | 0.000249 | 0.046198 |
| GAD1 | cg08863440 | -1.95509 | 0.00026 | 0.046198 |
| GAD1 | cg15993538 | -2.82688 | 0.000357 | 0.049139 |
| GFRA2 | cg15794228 | -2.12747 | 6.41 × 10^-5^ | 0.045957 |
| GNG2 | cg06243610 | 1.847592 | 0.000172 | 0.045957 |
| GPNMB | cg17274742 | 2.120824 | 0.000163 | 0.045957 |
| GPNMB | cg06616506 | 3.06965 | 0.000239 | 0.046198 |
| GRIN2D | cg15779837 | -5.1047 | 3.42 × 10^-5^ | 0.039106 |
| HAND2 | cg07681935 | -3.80832 | 0.000145 | 0.045957 |
| HAND2 | cg19669618 | -1.88799 | 0.000295 | 0.047277 |
| HMCN2 | cg18217252 | -2.44244 | 0.000213 | 0.046198 |
| IGSF9 | cg19354656 | 1.565236 | 0.000304 | 0.047277 |
| INHBA | cg09138133 | 2.885231 | 3.71 × 10^-5^ | 0.039106 |
| KLK10 | cg24512400 | 2.194267 | 0.000109 | 0.045957 |
| LSP1 | cg15899239 | 2.531132 | 0.000343 | 0.049139 |
| MS4A7 | cg09372819 | 2.698228 | 0.0002 | 0.046198 |
| MT1M | cg05581701 | 1.806797 | 0.000152 | 0.045957 |
| NEBL | cg21561038 | 2.248328 | 0.000282 | 0.046268 |
| NEBL | cg00822753 | 1.997342 | 0.000302 | 0.047277 |
| OTOP2 | cg04886642 | -1.77018 | 0.000127 | 0.045957 |
| OTOP2 | cg06353318 | -2.93026 | 0.000141 | 0.045957 |
| OTOP2 | cg12483561 | -2.46879 | 0.000152 | 0.045957 |
| P2RX2 | cg26620450 | -2.9181 | 0.00019 | 0.046198 |
| PADI2 | cg20929291 | 1.762424 | 0.000306 | 0.047277 |
| PCOLCE2 | cg11454468 | 3.932553 | 0.000348 | 0.049139 |
| PI16 | cg14420953 | 3.336876 | 5.93 × 10^-5^ | 0.045957 |
| PLCB1 | cg21844450 | -3.34351 | 9.64 × 10^-5^ | 0.039106 |
| PLCB1 | cg01431260 | -4.29642 | 3.37 × 10^-5^ | 0.039106 |
| PLCB1 | cg14805807 | -4.19027 | 3.66 × 10^-5^ | 0.039106 |
| PLCB1 | cg17510056 | -4.32475 | 8.60 × 10^-5^ | 0.045957 |
| PLCB1 | cg23657409 | -3.62742 | 0.00011 | 0.045957 |
| PLCB1 | cg27591117 | -2.99558 | 0.00026 | 0.046198 |
| PLCB1 | cg12496245 | -3.65615 | 0.000271 | 0.046198 |
| PLCB1 | cg23797439 | -1.88371 | 0.000386 | 0.049336 |
| PPM1H | cg01403748 | -2.2327 | 0.000168 | 0.045957 |
| PRIMA1 | cg06133145 | -3.49438 | 0.00013 | 0.045957 |
| PRKG2 | cg16023162 | -2.99455 | 0.000159 | 0.045957 |
| PRKG2 | cg26293000 | -2.45305 | 0.000177 | 0.046146 |
| RCN1 | cg26110474 | 2.563383 | 3.97 × 10^-5^ | 0.039106 |
| RCN1 | cg17047109 | 1.975469 | 0.000388 | 0.049336 |
| RELN | cg07450362 | 3.327158 | 0.000366 | 0.049139 |
| SCG2 | cg21269859 | 2.702038 | 4.11 × 10^-5^ | 0.039106 |
| SEMA6D | cg17875483 | -2.50577 | 0.000238 | 0.046198 |
| SEMA6D | cg10864878 | -3.22655 | 0.000268 | 0.046198 |
| SEMA6D | cg06624337 | -3.85498 | 0.000273 | 0.046198 |
| SEMA6D | cg18867004 | -3.21696 | 0.000364 | 0.049139 |
| SFRP2 | cg05874561 | -3.21511 | 1.61 × 10^-5^ | 0.039106 |
| SFRP2 | cg25645268 | -3.17453 | 3.42 × 10^-5^ | 0.039106 |
| SFRP2 | cg06549216 | -2.77927 | 3.89 × 10^-5^ | 0.039106 |
| SFRP2 | cg14063488 | -3.95746 | 7.95 × 10^-5^ | 0.045957 |
| SFRP2 | cg11354906 | -2.95387 | 0.000102 | 0.045957 |
| SFRP2 | cg25775322 | -2.33515 | 0.000156 | 0.045957 |
| SFRP2 | cg03202804 | -3.89748 | 0.000165 | 0.045957 |
| SFRP2 | cg00082664 | -2.68238 | 0.000173 | 0.045957 |
| SFRP2 | cg05774801 | -3.28542 | 0.000194 | 0.046198 |
| SFRP2 | cg10942078 | -1.93889 | 0.000266 | 0.046198 |
| SFRP2 | cg09788843 | -2.27511 | 0.000267 | 0.046198 |
| SFRP2 | cg23910835 | -2.59283 | 0.000307 | 0.047277 |
| SORCS1 | cg26554592 | -2.43885 | 0.000376 | 0.049336 |
| SP5 | cg12045337 | 2.896706 | 0.00036 | 0.049139 |
| SRD5A3 | cg14849597 | 4.377034 | 9.02 × 10^-6^ | 0.039106 |
| STOX2 | cg25882830 | -3.8165 | 2.78 × 10^-5^ | 0.039106 |
| STOX2 | cg02500300 | -3.22233 | 8.75 × 10^-5^ | 0.045957 |
| STOX2 | cg02071076 | -3.04669 | 0.000121 | 0.045957 |
| STOX2 | cg06367693 | -3.39994 | 0.000346 | 0.049139 |
| TLX1 | cg14861089 | -4.31297 | 9.14E-05 | 0.045957 |
| TLX1 | cg07494667 | -2.87475 | 0.000162 | 0.045957 |
| TLX1 | cg00299972 | -1.87645 | 0.000205 | 0.046198 |
| TLX1 | cg11196237 | -2.77963 | 0.000244 | 0.046198 |
| TLX1 | cg02450004 | -2.8682 | 0.000257 | 0.046198 |
| TLX1 | cg08908184 | -4.54462 | 0.000278 | 0.046198 |
| TLX1 | cg25741023 | -4.36191 | 0.000382 | 0.049336 |
| TOP1MT | cg20128188 | 2.576427 | 8.32 × 10^-5^ | 0.045957 |
| TOP1MT | cg11933583 | 1.829261 | 0.000123 | 0.045957 |
| TOP1MT | cg18063989 | 1.940686 | 0.000218 | 0.046198 |
| TRIM29 | cg16490778 | 2.085484 | 0.000246 | 0.046198 |
| VSTM2A | cg19868631 | -3.81463 | 0.000365 | 0.049139 |
| ZNRF3 | cg06866597 | 2.435419 | 5.78 × 10^-5^ | 0.045957 |
| ZSCAN18 | cg06243556 | -5.00648 | 9.10 × 10^-5^ | 0.045957 |
| ZSCAN18 | cg21653184 | -5.18022 | 0.000201 | 0.046198 |
| ZSCAN18 | cg23229395 | -3.08608 | 0.000309 | 0.047277 |
